# Enhanced 400-m sprint performance in moderately trained participants by a 4-day alkalizing diet: a counterbalanced, randomized controlled trial

**DOI:** 10.1186/s12970-018-0231-1

**Published:** 2018-05-31

**Authors:** Mirjam Limmer, Angi Diana Eibl, Petra Platen

**Affiliations:** 1 Department of Sports Medicine and Sports Nutrition, Ruhr-University Bochum, Gesundheitscampus Nord 10, 44801 Bochum, Germany; 2 Institute of Outdoor Sports and Environmental Science, German Sports University Cologne, Cologne, Germany

**Keywords:** Acid-base balance, Blood buffer capacity, Potential renal acid load, Anaerobic exercise performance

## Abstract

**Background:**

Sodium bicarbonate (NaHCO_3_) is an alkalizing agent and its ingestion is used to improve anaerobic performance. However, the influence of alkalizing nutrients on anaerobic exercise performance remains unclear. Therefore, the present study investigated the influence of an alkalizing versus acidizing diet on 400-m sprint performance, blood lactate, blood gas parameters, and urinary pH in moderately trained adults.

**Methods:**

In a randomized crossover design, eleven recreationally active participants (8 men, 3 women) aged 26.0 ± 1.7 years performed one trial under each individual’s unmodified diet and subsequently two trials following either 4 days of an alkalizing (BASE) or acidizing (ACID) diet. Trials consisted of 400-m runs at intervals of 1 week on a tartan track in a randomized order.

**Results:**

We found a significantly lower 400-m performance time for the BASE trial (65.8 ± 7.2 s) compared with the ACID trial (67.3 ± 7.1 s; *p* = 0.026). In addition, responses were significantly higher following the BASE diet for blood lactate (BASE: 16.3 ± 2.7; ACID: 14.4 ± 2.1 mmol/L; *p* = 0.32) and urinary pH (BASE: 7.0 ± 0.7; ACID: 5.5 ± 0.7; *p* = 0.001).

**Conclusions:**

We conclude that a short-term alkalizing diet may improve 400-m performance time in moderately trained participants. Additionally, we found higher blood lactate concentrations under the alkalizing diet, suggesting an enhanced blood or muscle buffer capacity. Thus, an alkalizing diet may be an easy and natural way to enhance 400-m sprint performance for athletes without the necessity of taking artificial dietary supplements.

## Background

The modern Western diet is considered to be a rather acidic diet [1, 2], as it includes acid-forming nutritional patterns like intake of high-protein, high-fat, and high-cholesterol animal products and a lack of base-forming intake of fruits and vegetables [3]. The resulting metabolic acidosis is associated with diseases of civilization such as obesity, diabetes, systemic hypertension, cardiovascular diseases and osteoporosis [1, 4]. Modifications to dietary composition reduce dietary acid loads and improve acid-base balance in humans [5–7]. In this context, new alkaline diets and supplements are being promoted and alkaline diets have gained popularity in the media over the last decade [2, 8].

The physiologic effects of dietary components on acid-base balance mainly involve the protein and mineral (e.g., potassium salts) contents of the diet, intestinal absorption rates of nutrients, and urinary acid excretion [9, 10]. The effects of ingested nutrients on acid-base balance can be quantified via the potential renal acid load (PRAL) [5, 11]. In general, meat, eggs, cheese, and cereal products promote systemic acidity (high-PRAL nutrients) while potatoes, vegetables, and fruits have the highest alkalizing potential (low-PRAL nutrients) [7, 12, 13]. The PRAL model is a calculation model based on the content of proteins, Cl^−^, PO4^3−^, SO4^2−^, Na^+^, K^+^, Ca ^2+^, and Mg ^2+^ [10]. PRAL can be calculated for each nutrient as follows: PRAL (mEq/100 g) = 0.49 × protein (g/100 g) + 0.037 × phosphorus (mg/100 g) - 0.021 × potassium (mg/100 g) - 0.026 × magnesium (mg/100 g) - 0.013 × calcium (mg/100 g) [6].

Besides the mentioned negative health effects of an acidizing diet, metabolic acidosis is further suggested to reduce exercise capacity during high-intensity exercise [10]. In that regard, it has been shown that ingestion of blood buffer modifying agents like sodium bicarbonate (NaHCO_3_) or sodium citrate can enhance high-intensity exercise performance [14, 15]. Bicarbonate is an extracellular buffer and ingestion of NaHCO_3_ rises the bicarbonate concentration ([HCO_3_^−^]) in extracellular fluids. Thus, the elevated [HCO_3_^−^] stimulates the lactate/H^+^ cotransporter, which leads to a greater efflux of H^+^ ions from the intracellular space into the extracellular fluid and allows buffering systems to remove H^+^ ions [15, 16]. The bicarbonate-induced enhanced buffering capacity seems to improve high-intensity anaerobic exercise performance [6].

The effects of a specific diet mainly containing alkalizing or acidizing nutrients on anaerobic performance, however, has not been examined sufficiently, even though nutrition influences acid base balance strongly [5, 7, 17]. Ball et al. [18] proposed that the ingestion of a diet low in carbohydrates and high in proteins and fat (high-PRAL diet) reduces the capacity to perform high-intensity exercise. The explanation of the authors, however, mainly focuses on carbohydrate metabolism and not on diet-induced metabolic acidosis. Further investigations did not find an influence of an alkalizing diet on anaerobic performance [6, 19, 20]. However, Rios Enriquez et al. [21] suggested an improvement in anaerobic exercise performance after an alkalizing diet for tests with a duration of 60 s to 2 min. In addition, a low-PRAL (alkalizing) diet improved the time to exhaustion during anaerobic exercise [7]. Further, an influence on blood and urinary pH, as well as blood bicarbonate values, has often been described when following either an acidizing or alkalizing diet [18, 21, 22].

Caciano et al. [7] concluded with the practical implication that the dietary manipulation of PRAL might benefit athletes and recommended a low-PRAL (alkalizing) diet for athletes training and competing in events heavily dependent on anaerobic metabolism, like 100–200 m swimming or 400–800 m running events. In addition, significant performance improvements relative to placebo trials were found for 400-m running events after oral NaHCO_3_ ingestion [23, 24]. Despite of these findings, in a recent review Applegate et al. [2] conclude that alkalizing diets do not demonstrate the same effects as alkalizing agents like NaHCO_3_ on buffering capacity and anaerobic performance.

In summary, recent studies suggest that the ingestion of a specific diet can modify blood buffer capacity, and that changes in blood buffering capacity can influence high-intensity anaerobic exercise performance. Therefore, the purpose of the present study was to investigate the influence of an alkalizing versus acidizing diet on 400-m sprint performance, maximum capillary blood lactate concentrations, blood gas parameters, and urinary pH in moderately trained young participants. We hypothesized that an alkalizing diet will enhance extracellular buffering capacity, and thus increase 400-m sprint performance.

## Methods

### Participants

A total of 16 young and nonspecifically trained participants volunteered to participate in the present study. Two participants withdrew from the study, due to busy time schedules. Three participants had incomplete data. Results presented are from the remaining 11 participants. The mean (± SD) age was 26.4 ± 1.8 y, with a mean height of 182.8 ± 6.9 cm and mean body mass of 82.0 ± 6.8 kg for male participants (*n* = 8) and 25.0 ± 1.0 y, 168.8 ± 1.4 cm, and 82.0 ± 6.8 kg for female participants (*n* = 3). All participants underwent a medical screening before entering the study. All participants were recreationally active and were familiar with sprinting activities. Participants were randomly selected students who volunteered from university level physical education classes, and who practiced various physical activities for ~ 12 h per week while pursuing their academic studies. Participants had to be healthy and without any injuries to the musculoskeletal system that could interfere with the execution of running. Individuals ingesting any nutritional supplements or following any specific diet in the 2 months prior to the initiation of the study were excluded. All participants gave their written informed consent prior to participation and all procedures were approved by the ethical committee of the Ruhr-University Bochum in accordance with the Declaration of Helsinki.

### Experimental design

**Fig. 1:**
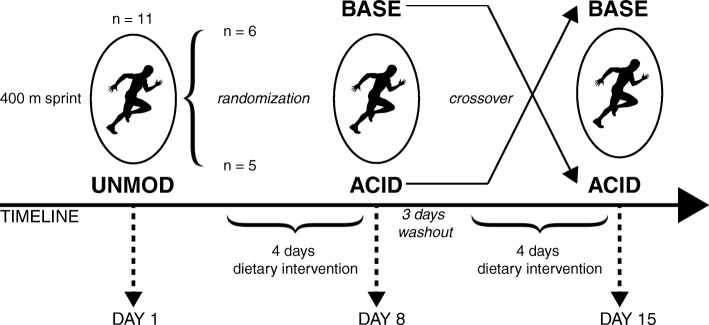
Experimental design

The study was designed as a randomized, single-blind, counterbalanced crossover trial (Fig. 1). Outcome assessors were blinded for group affiliation. Participants were informed about necessary modification of the diets to achieve high or low PRAL values, but not about expected influences of the diets and associated hypotheses. All participants performed three 400-m sprint exercise tests at intervals of 1 week on an outdoor tartan track. The three 400-m sprint exercise tests were performed in the morning at the same time of day. Before each 400-m performance test, the participants completed a pre-test warm-up, which included aerobic exercise and dynamic stretching. The first sprint trial served as habituation to the experimental protocol and was performed under each individual’s unmodified diet (UNMOD). Therefore, data of this first sprint trial was not included in statistical analyses. Four days before the two main trials, an acidizing (ACID) or alkalinizing (BASE) diet was followed in a randomized order. The first dietary intervention was followed by a 3-day washout phase with an unmodified diet before the second dietary intervention started in a crossover trial. Participants were instructed to abstain from alcohol and strenuous high-intensity exercise 24 h before each trial and compliance with these requests was verbally confirmed before each sprint trial.

### Dietary interventions

For each of the dietary interventions, a medical dietician provided specific instructions for the modification of the participants’ habitual diets to achieve high or low PRAL. A modified German version of the original PRAL food list published by the Institute for Prevention and Nutrition, Ismaning, Germany, was distributed to the participants [10]. Participants were instructed to make food choices and amounts ad libitum based on the respective PRAL values of foods. Participants were requested to document consumed foods and beverages on a food list during the dietary interventions. This enabled us to control the overall PRAL values. Focus was on food composition with respect to its influence on acid-base balance, while energy intake was not documented.

### Performance time

The overall 400-m performance time was measured using a stopwatch (Schütt PC-90, Schütt GmbH, Marburg, Germany). The timers were positioned right beside the finish line and were instructed to initiate their stopwatches on the start signal and to stop when the sprinters first foots pass the finish line. Each timer had several years of practice using a stopwatch and spent time learning the characteristics of their stopwatch used in this study. We used the same timer for each participant to attempt a higher interrater reliability of the hand-timing method, and timers were positioned in consistent timing positions.

### Urinary pH

Spontaneous urinary samples (at least 5 ml of urine) were collected before each sprint trial. Urinary pH values (pH_u_) were measured using Neutralit® pH-indicator strips pH 5.0–10.0 (Merck, Darmstadt, Germany). This parameter served as a surrogate marker to assure that the dietary intervention had been conducted successfully [25].

### Blood lactate

To assess blood lactate values, 20-μL capillary blood samples were collected from the earlobe before and every 2 min after the sprint trials, starting at minute 3 and continuing to minute 13 (3,5,7,9,11,13). Blood lactate measurements were conducted directly after the sprint trial (Biosen S-Line, EKF-diagnostic GmbH, Magdeburg, Germany). The maximum post-exercise lactate concentration (La_max_) was used for statistical analyses.

### Blood gas analysis

Capillary blood samples (100 μL) were taken from the hyperemized earlobe before and within the first minute after each sprint trial. Measurements were immediately conducted for blood gas parameters (Eschweiler Combiline, Eschweiler GmbH, Kiel, Germany). Parameters included oxygen and carbon dioxide partial pressure (*P*O_2_/*P*CO_2_), blood pH value (pH_b_), oxygen saturation (sO_2_), active blood bicarbonate concentration ([HCO_3_^−^]), and active base excess (BE). We further calculated the exercise-induced changes (Δ) of blood gas parameters pre- versus post-sprint. Pre-exercise and Δ values were used for statistical analyses.

### Statistical analysis

Data are presented as means ± standard deviations. The Shapiro-Wilk test was used to identify all departures from normal distribution. Paired sample *t*-tests were used to compare 400-m sprint performance times, La_max_, pre-exercise, post-exercise, and Δ blood gas parameters between the ACID and BASE conditions. In addition, paired sample *t*-tests were used to compare pre-exercise and post-exercise blood gas parameters. When variables were not normally distributed (pH_u_, pH_b_ pre-exercise, and *P*CO_2_ post-exercise), Wilcoxon tests were used to identify differences between the ACID and BASE conditions or pre- and post-exercise parameters. An a priori power calculation indicated that 11 participants were needed to detect significant differences in performance times, based on an estimated α level of 0.05 and a power of 95% (based on exercise performance enhancement results after an alkalizing diet from an earlier study [7]).The alpha level was set at *p* ≤ 0.05, and all analyses were conducted using SPSS 24 (IBM Corp., Armonk, NY, USA.).

## Results

**Fig. 2:**
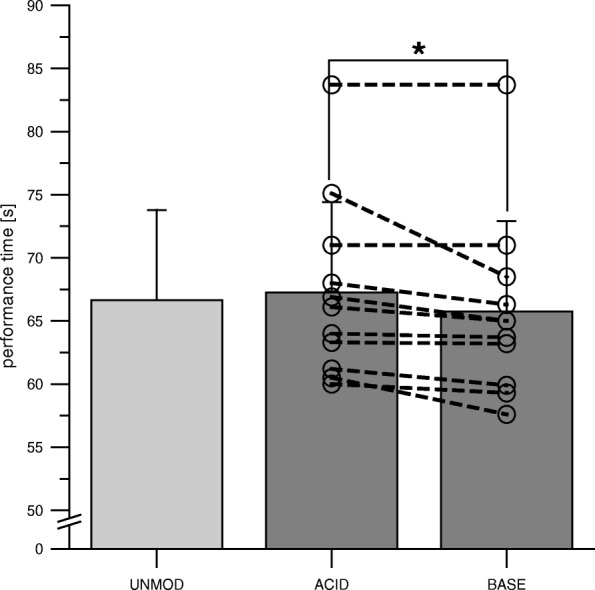
400 m running time for the acidizing (ACID) and alkalizing (BASE) diet trial. Data points represent individual values (○). Bar charts are means ± SD. See Methods for further details. *p = 0.026 compared with BASE

**Fig. 3:**
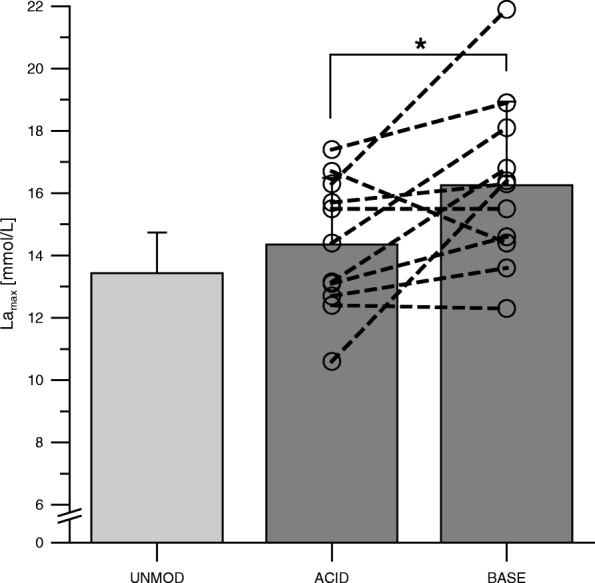
Maximal blood lactate values after a 400 m running event for the acidizing (ACID) and alkalizing (BASE) diet trial. Data points represent individual values (○). Bar charts are means ± SD. See Methods for further details. *p = 0.032 compared with BASE

**Fig. 4:**
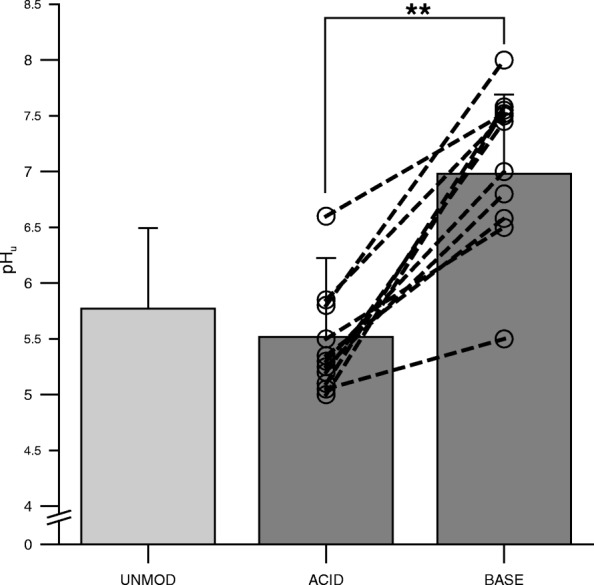
Urinary pH values after four days of the acidizing (ACID) and alkalizing (BASE) diet. Data points represent individual values (○). Bar charts are means ± SD. See Methods for further details. *p = 0.007 compared with BASE

The overall 400-m performance time was 2.3% faster in the BASE trial (65.8 ± 7.2 s) compared with the ACID trial (67.3 ± 7.1 s; *p* = 0.026) (Fig. 2). La_max_ was significantly higher for the BASE trial (16.3 ± 2.7 mmol/L) compared with the ACID trial (14.4 ± 2.1 mmol/L; *p* = 0.032) (Fig. 3). Urine pH was significantly higher after the BASE diet compared with the ACID diet (BASE: 7.0 ± 0.7, ACID: 5.5 ± 0.7; *p* = 0.001) (Fig. 4). Data of the UNMOD trial are shown in Figs. 1, 2, and 3, but were not included in statistical analyses.

Post-exercise values were significantly lower compared with pre-exercise values for blood gas parameters pH, [HCO_3_^−^], BE, and sO_2_, but not for *P*O_2_ and *P*CO_2_during the BASE trial (pH: *p* < 0.000, [HCO_3_^−^]: *p* < 0.000, BE: *p* < 0.000, sO_2_: *p* = 0.004, *P*O_2_: *p* = 0.712, *P*CO_2_: *p* = 0.087) and during the ACID trial (pH: *p* = 0.003, [HCO_3_^−^]: *p* < 0.000, BE: *p* < 0.000, sO_2_: *p* = 0.006, *P*O_2:_*p* = 0.836, *P*CO2: *p* = 0.182) (Table 1). There were no significant differences in any blood gas parameter between groups for pre-exercise, post-exercise, and Δ values (Table 1). There was, however, a non-significant tendency for a higher pre-exercise [HCO_3_^−^] value in the BASE compared with the ACID trial (*p* = 0.063).

**Table 1:** Pre- and post- 400 m sprint values and sprint-induced changes (Δ) of oxygen and carbon dioxide partial pressure (*P*O_2_ / *P*CO_2_), active blood bicarbonate concentration ([HCO_3_^−^]), active base excess (BE), oxygen saturation (sO_2_), and blood pH value (pH_b_) after 4 days of an acidizing (ACID) or alkalizing (BASE) dietary intervention

		ACID	BASE	*p***-**value
*P*O_2_ [mmHg]	pre - sprint	116.3 ± 26.4	118.7 ± 26.2	0.823
	post - sprint	118.1 ± 19.1	115.5 ± 20.1	0.789
	Δ sprint	1.8 ± 28.3	−3.3 ± 28.6	0.653
*P*CO_2_ [mmHg]	pre - sprint	35.1 ± 3.5	36.5 ± 4.9	0.387
	post - sprint	31.2 ± 7.5	33.3 ± 5.5	0.385
	Δ sprint	−3.9 ± 9.3	− 3.3 ± 5.7	0.732
[HCO_3_^−^] [mmol/L]	pre - sprint	24.3 ± 2.0	25.9 ± 2.8	0.063
	post - sprint	12.8 ± 3.3	13.0 ± 2.4	0.818
	Δ sprint	−11.4 ± 3.8	−12.9 ± 2.3	0.155
BE [mmol/L]	pre - sprint	1.30 ± 2.39	2.32 ± 2.20	0.184
	post - sprint	−13.56 ± 3.39	−13.87 ± 3.27	0.780
	Δ sprint	−14.86 ± 3.82	−16.19 ± 2.88	0.310
sO_2_ [%]	pre - sprint	98.4 ± 0.9	98.5 ± 0.6	0.603
	post - sprint	97.0 ± 1.2	96.8 ± 1.5	0.668
	Δ sprint	−1.4 ± 1.3	−1.8 ± 1.6	0.449
pH_b_	pre - sprint	7.46 ± 0.05	7.47 ± 0.03	0.373
	post - sprint	7.24 ± 0.04	7.22 ± 0.07	0.294
	Δ sprint	−0.22 ± 0.06	−0.24 ± 0.07	0.347

Data is presented as mean ± standard deviation of the mean. No significant differences between groups were found using paired sampled *t*-tests or Wilcoxon tests if variables were not normally distributed (pH_b_ pre-sprint and *P*CO_2_ post-sprint). *n* = 11

We also examined for a potential confounding effect by comparing the first and second sprint trials (independent of the dietary intervention) using a paired sample *t*-test. However, there was no difference between the two trials (*p* = 0.606), suggesting that there was no training effect that may have negatively influenced our test results.

## Discussion

In the present study we investigated the influence of a 4-day alkalizing versus acidizing diet on 400-m sprint performance and associated physiological markers in moderately trained young participants. Our major finding is that the alkalizing diet results in an improved 400-m sprint time, higher blood lactate, but unchanged blood pH values compared with the acidizing diet.

### Urinary pH

In the present investigation, we found significantly higher urine pH values for the BASE trial (7.0 ± 0.7) compared with the ACID trial (5.5 ± 0.7). Thus, we assume that the dietary intervention was conducted successfully because a urine pH of ≥7.0 is expected for successful low-PRAL diets and ≤ 6.0 for high-PRAL diets [7, 25].

### Sprint performance

To the best of our knowledge, this is the first study to estimate the influence of acid- and alkaline-forming nutrition on anaerobic exercise performance with high applicability for a sport discipline. There are a number of studies estimating the effects of a dietary acid load on anaerobic exercise performance using exercise tests exclusive to cycling or treadmill running [2, 6, 7, 18, 21, 22]. However in a recent review, Applegate et al. [2] postulated a lack of studies examining different exercise intensities and measures of performance regarding an alkalizing diet. Additionally, Caciano et al. [7] recommended dietary manipulation of PRAL for sporting events where performance is limited as result of acidosis, like 100–200-m swimming or 400–800-m running events. Sprint performance for 400-m trials has already been suggested to improve after ingestion of NaHCO_3_ [16, 23], but this has not been investigated for an alkalizing diet. Therefore, based on the presumption that an alkalizing, low-PRAL diet increases systemic alkalinity and blood buffer capacity, we hypothesized that an alkalizing diet also increases 400-m sprint performance [7, 21]. Indeed, in the recent study, 400-m sprint performance time was significantly lower for the BASE trial compared with the ACID trial, indicating that sprint performance was enhanced after consuming mainly low-PRAL nutrients for 4 days prior to the sprint test. However, the sprint performance enhancement was only 2.3% in our study and less pronounced compared with the 21% increase of exercise performance in the recent literature [7]. We consider the difference in the performance tests as the main reason for this incongruence. Whereas we estimated performance as the run time for a fixed distance (time-trial test), Caciano et al. [7] assessed anaerobic performance as time-to-exhaustion while running on a treadmill with an individually defined and fixed speed. Open-ended protocols with time-to-exhaustion introduce larger variability in performance output than distance-based performance tests, mainly because of motivational and mental aspects [23, 26]. Therefore, we assume that a lower but more constant performance improvement is to be expected for time-trial tests, such as a 400-m sprint trial, compared with time-to-exhaustion tests after an alkalizing low-PRAL diet [26]. Thus, 400-m sprint performance time was enhanced after the low-PRAL diet, though the use of hand timing to measure the 400-m time trial is one of the limitations of this study. The most precise and preferred method of timing is by electronic methods because of the absolute errors associated with hand timing [27, 28]. For example, variations among hand timers are likely to occur [28]. Additionally, hand timing produces a faster sprint time than electronic timing [28, 29], and a correction factor of 0.2 s has traditionally been used for hand timing [30]. On the other hand, small mean errors (0.04–0.05 s) and very high correlation values (ICC 0 0.99) have been observed between hand timing and electronic timing, which indicates that hand timing produces consistent sprint times for the same hand timer [28, 31]. Hand timing was the only method available to be used in evaluating sprint times in this investigation. Therefore, we decided to apply several measurement strategies supposed by Mayhew et al. [28] in order to minimize problems with this method. We used the same timer for each participant to attempt a higher interrater reliability of the hand-timing method, and timers were positioned in consistent timing positions perpendicular to the finish line. Each timer was proficient in the use of a stopwatch and spent time learning the characteristics of the stopwatch used in this study. Furthermore, we asked the tester to initiate the timing with the index finger and not with the thumb, as it was previously reported that the most reliable and objective handheld stopwatch times are achieved when the timer uses the index finger to operate the stopwatch [28, 32]. We think that these strategies reduced the errors associated with hand timing and resulted in consistent sprint times within the present study.

### Blood lactate and blood gas analysis

We found significantly lower values for the blood gas parameters pH, [HCO_3_^−^], and BE post-exercise compared with pre-exercise for both dietary interventions (BASE and ACID). This indicates a profound exercise-induced metabolic acidosis after 400-m sprint trials for both conditions.

Further, we found higher maximum post-exercise lactate concentrations after 400-m sprint performance during the BASE trial compared with the ACID trial. Robergs et al. [33] state lactate production during intense exercise more as a consequence rather than a cause of cellular conditions that cause acidosis. However, these authors conclude that lactate is still a good indirect marker for cellular metabolic conditions that induce metabolic acidosis because increased lactate production coincides with acidosis [33]. Therefore, higher blood lactate values during the BASE trial within the recent study in combination with the improved 400-m sprint time (i.e., more energy demand per time unit) might indicate a higher efflux of H^+^ ions from the muscle cell across the interstitial space and into the venous circulation, creating a more severe metabolic acidosis. However, we found no differences in blood pH between BASE and ACID within the recent study. The lack of differences in blood pH between both dietary interventions is probably a result of the higher blood buffer capacity because of high [HCO_3_^−^] concentrations associated with an alkalizing diet [34].

An augmentation of the [HCO_3_^−^] concentration as well as an increased blood pH can both be found after sodium bicarbonate supplementation [15, 16, 24, 35]. Unfortunately, the alkalizing or acidizing dietary intervention did not result in significant differences for any of the blood gas parameters within this study (Table 1). However, we found a slight tendency towards higher [HCO_3_^−^] values following a low-PRAL diet for 4 days (Table 1). It has been suggested in recent literature that alkalizing diets are unlikely to produce the same changes in buffer capacity compared with alkalizing ergogenic aids and that consumption of low-PRAL diets produces only a slight, but insufficient alkaline environment to enhance buffer capacity [2, 13]. Our study, however, clearly indicates that total buffer capacity must have been increased after a 4-day alkalizing diet because we did not find changes in blood pH but increased blood lactate concentrations and faster 400-m sprint times. Therefore, we assume that the non-significant tendencies towards [HCO_3_^−^] and BE values (Table 1) indicate a higher buffer capacity after an alkalizing diet and might be more apparent when testing a larger sample size or longer duration of the dietary intervention.

### Practical applications

First, a large inter-subject variation in PRAL from normal Western diets exists among athletes [6, 12]. Considering this individual variability, sprint athletes and coaches should be encouraged to undergo a dietary assessment, including urine pH measurements, before an alkalizing diet is applied. Fasted morning urine pH can be monitored for assessment and during the low-PRAL dietary intervention to confirm that the diet adequately alters dietary acid load [7]. Urinary pH values of ≥7.0 may be interpreted as a successful low-PRAL diet and values of ≤6.0 as high-PRAL diets [7, 25]. However, individual variability must be considered when interpreting urine pH values.

Second, when consuming alkalizing diets, it is often suggested to obtain the PRAL by increasing consumption of fruits and vegetables and minimizing consumption of meats and grains [12]. Based on this advice, a caloric deficit during consuming alkalizing diets is reported [21]. Conclusively, especially for sprint athletes, the higher energy demands and needs for dietary protein and carbohydrate sources, of which increase the PRAL, may make it difficult to realize an alkalizing diet [6, 12, 13]. Regarding this problem, we highly advise the additional use of mineral waters rich in bicarbonate to simplify the realization of an alkalizing diet [13, 36, 37]. Additionally, consumption of carbohydrate-rich fruits and vegetables, such as fresh and dried fruits, fruit juices, and potatoes, should be encouraged [7]. A food diary might be used to control the amount of foods eaten during a low-PRAL diet period. Food diaries can be analyzed for energy and macronutrient intake as well as for calculation of the overall PRAL per day. The lack of food diaries as well as analyses of PRAL values, energy intake and macronutrient content is another limitation of the present study. We asked our participants to report the foods eaten within each day of the dietary interventions, however, we did not collect amount of foods. Thus, we assume that the dietary interventions had been conducted successfully, because diaries mainly contained of vegetables and fruits during the low-PRAL diet and of grain and dairy products during the high-PRAL diet. However, we were not able to analyze energy intake or macro- and micronutrient content of the foods. Therewith we cannot report about an influence of carbohydrate (CHO) content on sprint performance, which has already been investigated [38, 39]. Couto et al. [38] showed that a high CHO diet induced higher CHO oxidation rates and increased running speed in 400-m sprints. Although, we do not think that high CHO intake might have influenced the 400-m sprint performance for the low-PRAL trial positively in this study. We presume low-PRAL dietary recommendations for that, because recommendations limit the use of carbohydrate sources (grains, e.g. bread or pasta) as they increase the PRAL. Therewith, these dietary recommendations lead more to caloric deficits during consuming alkalizing diets than to CHO loading [21].

In addition, some authors suggest a responder/non-responder phenomenon to the ergogenic potential of bicarbonate supplementation, with a tendency for highly trained athletes to show higher effects than untrained individuals [15, 40]. Gastrointestinal (GI) discomfort is dosage-dependent for NaHCO_3_ ingestion and GI discomfort may negatively affect sprint performance [15, 41]. Neither has been reported for a low-PRAL diet so far; however, we highly recommend a test phase for each athlete during a non-competitive training period before changing the usual diet during competitions to inhibit discomfort from the dietary intervention.

Moreover, alkalizing low-PRAL diets lead to a chronic alkalotic state and, therefore, might be compared with chronic use of NaHCO_3_ in some respects. There is evidence that despite the acute effects of bicarbonate ingestion on anaerobic performance in competitive situations, chronic use of NaHCO_3_ in combination with specific training may lead to aerobic adaptions. Chronic NaHCO_3_ ingestion coupled with high intensity training may further influence mechanisms associated with muscle force production or rapid force-generating capacity [16]. The authors conclude that there is a lack of investigation into the possible effects of chronic adaptions to training in an alkalotic state. Regarding alkalizing dietary recommendations for sprint athletes, which are mainly chronic interventions, further research in this field is needed to clarify these training effects in a chronic alkalotic state.

### Limitations of the study

A limitation of our study was the use of hand timing, which introduces a certain level of inaccuracy in measuring 400-m sprint performance. Previous studies have shown that electronic timing is the more precise and preferred method of timing [27, 28]. To reduce the potential errors associated with hand timing, we applied several measurement strategies, including using the same timer for each participant, and consistent positioning of the timers perpendicular to the finish line [28]. However, future studies should consider electronic timing when investigating 400-m sprint performance in a small sample size. Another limitation of the study is the lack of quantitative information on food intake to allow for detailed analyses of PRAL values, energy intake, and macronutrient content. The participants were asked to provide daily qualitative food reports. However, the total amount and dietary composition were not controlled. The food reports conducted in our study mainly contained vegetables and fruits during the low-PRAL diet, and grain and dairy products during the high-PRAL diet. The application of extensive nutritional analyses in future studies is required to support the validity of our findings. Finally, there was a small sample size (*n* = 11) in our study, which resulted in wide confidence intervals and high *p*-values. Nevertheless, despite this limitation, a significant effect of dietary intervention was observed.

## Conclusion

The present study was the first to examine the effects of a short-term alkalizing or acidizing diet on 400-m sprint performance in moderately trained participants. Our data suggest that it is possible to improve 400-m performance by consuming alkalizing (low-PRAL) natural foods and beverages, without the ingestion of dietary supplements like NaHCO_3_ or sodium citrate. Additionally, we found higher blood lactate but unchanged blood pH values for the alkalizing trial compared with the acidizing trial. Thus, an alkalizing diet may be an easy and natural way to enhance the tolerance towards exercise-induced alkalosis for athletes without the necessity of taking artificial dietary supplements.

## Abbrevations

[HCO_3_^−^]: Active blood bicarbonate concentration
ACID: Acidizing diet
BASE: Alkalizing diet
BE: Active base excess
CHO: Carbohydrate
cm: Centimeter
kg: Kilogram
La_max_: Maximum post-exercise blood lactate concentration
min: Minutes
NaHCO_3_: Sodium bicarbonate
*P*CO_2_: Carbon dioxide partial pressure
pH_b_: Blood pH
pH_u_: Urinary pH
*P*O_2_: Oxygen partial pressure
PRAL: Potential renal acid load
s: Seconds
SD: Standard deviations
sO_2_: Oxygen saturation
UNMOD: Unmodified diet
